# Supplementary material for: Six-year (2016–2022) longitudinal patterns of mental health service utilization rates among children developmentally vulnerable in kindergarten and the COVID-19 pandemic disruption
Source: PLOS Digit Health. 2024 Sep 17;3(9):e0000611. doi: 10.1371/journal.pdig.0000611 (PMC11407640; doi:10.1371/journal.pdig.0000611)
Supplement: S1 Table — (DOCX) [file pdig.0000611.s001.docx]

**Table S1**. List of ICD 9 and 10 codes for mental health conditions.

|  | ICD-9 (used for physicians’ office visits) | ICD-10 (used for emergency visits and hospitalizations) |
| --- | --- | --- |
| All mental health | '290','291','292','293','294','295','296','297','298','299','300','301','302','303','304','305','306','307','308','309','310','311','312','313','314','315','316','317','318','319', '331','341' | ‘F’,'G30','X60','X61','X62','X63','X64','X65','X66','X67','X68','X69', 'X70','X71','X72','X73','X74','X75','X76','X77','X78','X79','X80','X81','X82','X83','X84','Y87','T51', 'G210', 'G211', 'G240', 'G251', 'G259', 'T740', 'T741', 'T742', 'T509', 'Y870', 'Z004', 'Z046' |
| Anxiety | '300','308','300.1','300.4','300.5','300.6','300.7','300.8','300.9' | 'F40','F41','F42','F43','F93' |
| Mood disorders | '296','311','300.4' | 'F30','F31','F32','F33','F34','F38','F39' |
| ADHD | '314' | ‘F90' |
