# Supplementary material for: Six-year (2016–2022) longitudinal patterns of mental health service utilization rates among children developmentally vulnerable in kindergarten and the COVID-19 pandemic disruption
Source: PLOS Digit Health. 2024 Sep 17;3(9):e0000611. doi: 10.1371/journal.pdig.0000611 (PMC11407640; doi:10.1371/journal.pdig.0000611)
Supplement: S2 Table — (DOCX) [file pdig.0000611.s002.docx]

**Table S2**. Results of linear regression models for domain-specific analysis of all utilization.

|  |  | **Office visits** | | **Emergency department visits** | | **Hospitalizations** | |
| --- | --- | --- | --- | --- | --- | --- | --- |
| **Domain** | **Variable** | **Beta** | **P-value** | **Beta** | **P-value** | **Beta** | **P-value** |
| General knowledge (CG) | Vulnerability | 762.6 | 0.004 | not significant | | not significant | |
| Emotional maturity (EM) | Vulnerability | 676.7 | 0.006 | 129.8 | 0.009 | 6.2 | 0.040 |
| Language and cognitive development (LC) | Vulnerability | 780.5 | 0.004 | 93.8 | 0.040 | 10.8 | 0.040 |
| Physical health and well-being (PH) | Vulnerability | 751.6 | 0.004 | 122.4 | 0.015 | 12.9 | 0.021 |
| Social competence (SOC) | Vulnerability | 794.1 | 0.004 | 117.1 | 0.018 | 7.6 | 0.040 |

*Note*: the variable Sex and the interaction term Vulnerability*Sex was not significant in any domain.
