# Supplementary material for: Six-year (2016–2022) longitudinal patterns of mental health service utilization rates among children developmentally vulnerable in kindergarten and the COVID-19 pandemic disruption
Source: PLOS Digit Health. 2024 Sep 17;3(9):e0000611. doi: 10.1371/journal.pdig.0000611 (PMC11407640; doi:10.1371/journal.pdig.0000611)
Supplement: S3 Table — (DOCX) [file pdig.0000611.s003.docx]

**Table S3**. Results of linear regression models for domain-specific analysis of mental health-related utilization.

|  |  | **Office visits** | | **Emergency department visits** | | **Hospitalizations** | |
| --- | --- | --- | --- | --- | --- | --- | --- |
| **Domain** | **Variable** | **Beta** | **P-value** | **Beta** | **P-value** | **Beta** | **P-value** |
| General knowledge (CG) | Vulnerability | 306.2 | 0.002 | - | - | not significant | |
|  | Vulnerability*Sex | not significant | | 4.1 | 0.020 | not significant | |
| Emotional maturity (EM) | Vulnerability*Sex | 196.4 | 0.012 | not significant | | not significant | |
| Language and cognitive development (LC) | Vulnerability | 373.6 | 0.002 | 3.6 | 0.046 | 2.6 | 0.046 |
| Physical health and well-being (PH) | Vulnerability | 427.9 | 0.002 | not significant | | not significant | |
|  | Sex | 194.1 | 0.002 | not significant | | not significant | |
| Social competence (SOC) | Vulnerability | - | - | - | - | 2.7 | 0.020 |
|  | Vulnerability*Sex | 191.6 | 0.005 | 8.4 | 0.002 | not significant | |

*Note*: Non-significant variables are omitted in this table.
