# Supplementary material for: Six-year (2016–2022) longitudinal patterns of mental health service utilization rates among children developmentally vulnerable in kindergarten and the COVID-19 pandemic disruption
Source: PLOS Digit Health. 2024 Sep 17;3(9):e0000611. doi: 10.1371/journal.pdig.0000611 (PMC11407640; doi:10.1371/journal.pdig.0000611)
Supplement: S4 Table — (DOCX) [file pdig.0000611.s004.docx]

**Table S4**. Results of linear regression models for domain-specific analysis of specific mental health disorders.

|  |  | **Mood disorder** | | **Anxiety** | | **ADHD** | |
| --- | --- | --- | --- | --- | --- | --- | --- |
| **Domain** | **Variable** | **Beta** | **P-value** | **Beta** | **P-value** | **Beta** | **P-value** |
| General knowledge (CG) | Vulnerability | not significant | | not significant | | 152.8 | 0.002 |
|  | Sex (M) | not significant | | not significant | | 193.9 | 0.002 |
| Emotional maturity (EM) | Vulnerability*Sex | not significant | | not significant | | 158.5 | 0.024 |
| Language and cognitive development (LC) | Vulnerability | not significant | | not significant | | 160.6 | 0.005 |
|  | Sex (M) | not significant | | not significant | | 188.1 | 0.002 |
| Physical health and well-being (PH) | Vulnerability | not significant | | 42.2 | 0.040 | 202 | 0.002 |
|  | Sex | not significant | | not significant | | 173.9 | 0.002 |
| Social competence (SOC) | Vulnerability*Sex | not significant | | not significant | | 138.1 | 0.027 |

*Note*: Non-significant variables are omitted in this table.
