# Supplementary material for: Six-year (2016–2022) longitudinal patterns of mental health service utilization rates among children developmentally vulnerable in kindergarten and the COVID-19 pandemic disruption
Source: PLOS Digit Health. 2024 Sep 17;3(9):e0000611. doi: 10.1371/journal.pdig.0000611 (PMC11407640; doi:10.1371/journal.pdig.0000611)
Supplement: S1 Fig — (DOCX) [file pdig.0000611.s005.docx]

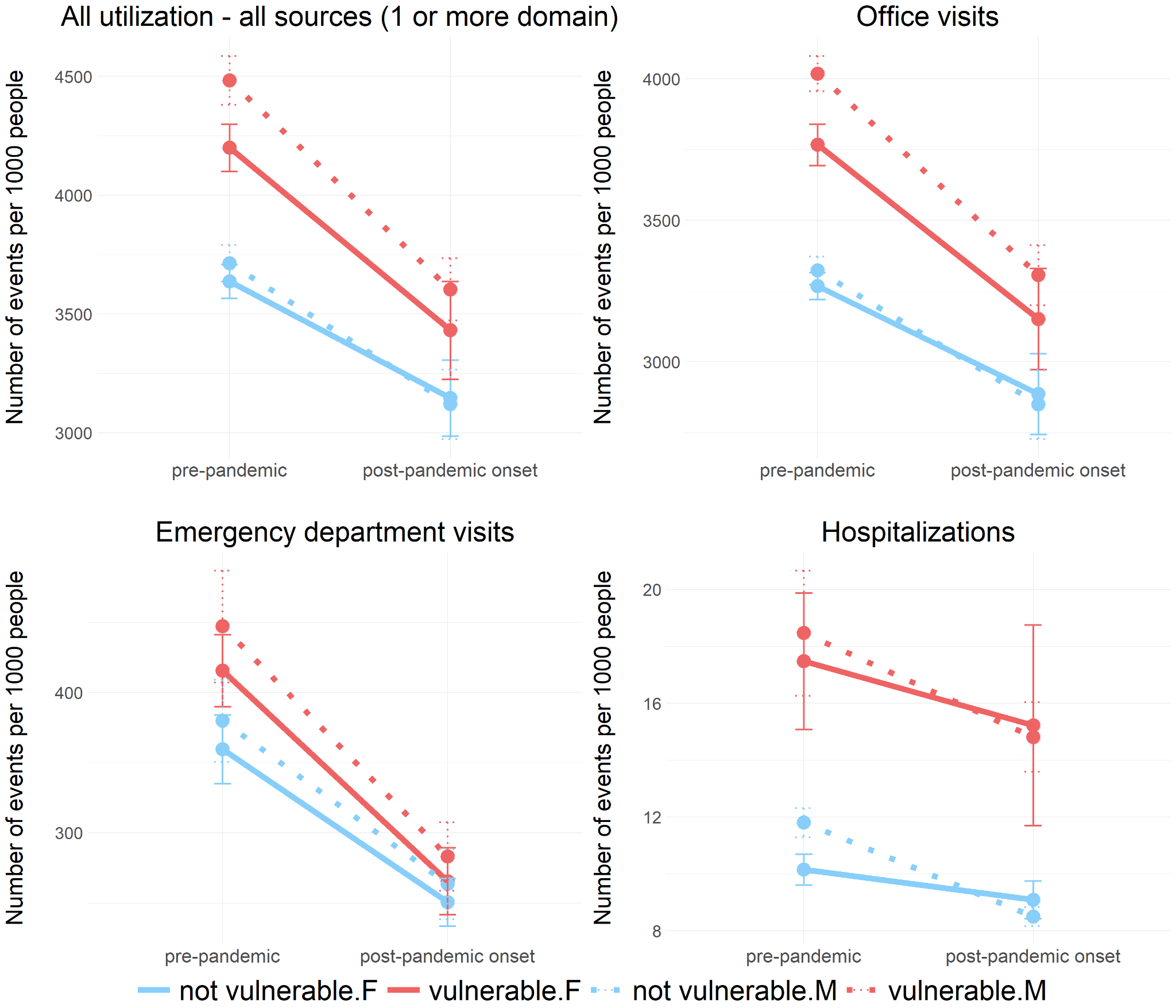


**Figure S1**. Comparison of health services utilization between the pre (2016-2019) and post (2020-2022) pandemic onset periods.

*Error bar indicates standard error.*
