# Supplementary material for: Six-year (2016–2022) longitudinal patterns of mental health service utilization rates among children developmentally vulnerable in kindergarten and the COVID-19 pandemic disruption
Source: PLOS Digit Health. 2024 Sep 17;3(9):e0000611. doi: 10.1371/journal.pdig.0000611 (PMC11407640; doi:10.1371/journal.pdig.0000611)
Supplement: Attachment — Submitted filename: RESPON~1.DOC [file pdig.0000611.s007.DOC]

**Reviewer #1:**

Important topic and good contribution from the research to help address and design interventions / preventions in more targeted population taking in consideration gender and pre vulnerability.

Answer: We appreciate you taking the time to read and review the article.

**Reviewer #2:**

A. General comment:

This is a good study which I recommend for publication following the correction of minor errors highlighted below;

Answer: We thank the reviewer for the comment.

B. Specific comments

Query 1: under methods subsection participants

Line 104, the authors need to recheck the percentage quoted as eligible population included in the study (61.1%). This should be 23496/38358 = 61.2% from the figure presented.

Answer: Thank you for noticing this mistake. The percentage was changed to 61.2% in the manuscript.

Query 2: under methods subsection participants

Line 106: The authors need to be consistent with a referencing style. Hence, replace the Talarico eta al 2023 with Vancouver referencing (numeric referencing style).

Answer: The reference was replaced with Vancouver style (‘Talarico et al., 2023’ to ‘16’).

Query 3: under Result section

Line 171-172: ‘Among the 23494 children, there were 2445 (29.7%) females’. This frequency and percentage quoted need to be rechecked by the authors. Overall (i.e vulnerable and non-vulnerable), Females constituted 48% (11289/23494) as presented in Table 1. Likewise, the mean age should be rechecked. Kindly recheck these and make necessary adjustment or provide clarity to the frequency and percentages earlier quoted above.

Answer: Thank you for noticing the mistake in the number and percentage of female children in the text. The mean age was double-checked and the numbers in the table are correct. The paragraph now reads: “The mean age of vulnerable and non-vulnerable children was 5.63 and 5.70, respectively. Among the 23,494 children, 11,289 (48.1%) were females and 6,702 were classified as vulnerable in one or more development domains.”

Query 4: under Result section

Line 186, Table 2: The Column Letter ‘N’ and the Row ‘N’ need to be delineated and well defined. The authors defined the ‘N’ and ‘Y’ on the row at the bottom of the table but this is not so for that on the Column. I suggest the authors use smaller ‘n’ for that of the column and define it at the bottom of the table for clarity.

Answer: ‘N’ was replaced with ‘n’ for clarity in both tables 1 and 2. The definition was added at the bottom of the tables as ‘n = number (sample size) or number of events (utilization)’.

**Reviewer #3:**

The paper highlighted the importance of the escalating global prevalence of mental health disorders in children and adolescents as well as the impact of early life adversities on mental health.

Specifically, the study explored the utilization patterns of mental health-related services among 6–12-year-old children in Alberta, with known kindergarten teacher-rated developmental vulnerability status, prior to and after the onset of the COVID-19 pandemic.

The findings showed that developmentally vulnerable children demonstrated higher engagement with mental health-related services compared to their non vulnerable counterparts across the whole time period studied, underlining the demand for targeted interventions and comprehensive support strategies for vulnerable populations, and underscoring the imperative of equitable access to mental health services.

The paper is therefore relevant and contributes to the body of knowledge.

The paper generally followed the structure of a scientific article.

The introduction was appropriate and adequately provided enough background knowledge about the subject matter.

The authors spent talked about the children excluded from the study and even made some comparison with those included in the study. This should not be necessary since those children were excluded from this study. If the authors want to write another paper on such comparison, it will be a good idea.

Answer: We thank the reviewer for the comment.

-There was wrong referencing style while talking about the publication on the inclusion and exclusion criteria. i.e (Talarico et al., 2023).

Answer: The reference was replaced with Vancouver style (‘Talarico et al., 2023’ to ‘16’).

-Some of the figures and percentages in the results were difficult to understand. The figure, 2,445 is reported as 29.7% females. However, 2445 is not 29.7% of 23,494. The authors should explain and clarify the figures with the percentage.

Answer: Thank you for noticing the mistake in the number and percentage of female children in the text. The mean age was double-checked and the numbers in the table are correct. The paragraph now reads: “The mean age of vulnerable and non-vulnerable children was 5.63 and 5.70, respectively. Among the 23,494 children, 11,289 (48.1%) were females and 6,702 were classified as vulnerable in one or more development domains.”

-The figures should follow the narratives so that it can be easy to relate what is read to the figure for easy understanding.

Answer:

We double-checked the placement of the figures and made sure they followed the narratives. Due to the journal requirement, the figures must be uploaded separately. We added where the figures should be in the main text.

-The tables are not clear enough to understand. The table needs to be better presented with the columns and rows clearly delineated to clearly understand the information presented even by a layman.

Answer: ‘N’ was replaced with ‘n’ for clarity in both tables 1 and 2 so it is not mistaken with ‘N = no’. The definition was added at the bottom of the tables as ‘n = number (sample size) or number of events (utilization)’. Table formatting was also edited for clarity: 1) bottom border line was added between the first and second row to separate the column name from the data; 2) The first row is now in bold.

-Overall the study is a good one and has contributed to the body of knowledge.

Answer: We appreciate you taking the time to read and review the article.
